# Teneurins: Role in Cancer and Potential Role as Diagnostic Biomarkers and Targets for Therapy

**DOI:** 10.3390/ijms22052321

**Published:** 2021-02-26

**Authors:** Giulia Peppino, Roberto Ruiu, Maddalena Arigoni, Federica Riccardo, Antonella Iacoviello, Giuseppina Barutello, Elena Quaglino

**Affiliations:** Molecular Biotechnology Center, Department of Molecular Biotechnology and Health Sciences, University of Torino, Via Nizza 52, 10126 Torino, Italy; giulia.peppino@unito.it (G.P.); roberto.ruiu@unito.it (R.R.); maddalena.arigoni@unito.it (M.A.); federica.riccardo@unito.it (F.R.); antonella.iacoviello@edu.unito.it (A.I.); giuseppina.barutello@unito.it (G.B.)

**Keywords:** teneurins, cancer, tumor progression, metastasis

## Abstract

Teneurins have been identified in vertebrates as four different genes (TENM1-4), coding for membrane proteins that are mainly involved in embryonic and neuronal development. Genetic studies have correlated them with various diseases, including developmental problems, neurological disorders and congenital general anosmia. There is some evidence to suggest their possible involvement in cancer initiation and progression, and drug resistance. Indeed, mutations, chromosomal alterations and the deregulation of teneurins expression have been associated with several tumor types and patient survival. However, the role of teneurins in cancer-related regulatory networks is not fully understood, as both a tumor-suppressor role and pro-tumoral functions have been proposed, depending on tumor histotype. Here, we summarize and discuss the literature data on teneurins expression and their potential role in different tumor types, while highlighting the possibility of using teneurins as novel molecular diagnostic and prognostic biomarkers and as targets for cancer treatments, such as immunotherapy, in some tumors.

## 1. Introduction

Teneurins belong to a conserved family of transmembrane proteins that are involved in cell–cell and cell–extracellular matrix interactions, with a pivotal role in embryonic development and nervous system function. Teneurin homologs have been functionally implicated in axon guidance [1], neurite outgrowth [1,2], and transcriptional regulation [3,4,5], as well as in cell proliferation and adhesion [1]. Teneurin genes were first described in vertebrates in 1998 [6] as orthologs of the Ten-m/odd Oz (Odz) genes that had previously been independently identified in Drosophila by two groups using two different strategies [7,8]. Baumgartner and colleagues discovered the Ten-m gene by screening the Drosophila genome with a Tenascin-a probe that covers its epidermal growth factor (EGF)-like domain [7], while Levine and colleagues described the Odz gene via a tyrosine phosphorylation-based screening [8]. Initially, Ten-m/Odz proteins were defined as pair-rule genes involved in Drosophila segmentation, and were mainly expressed in the central nervous system and heart [7,8]. Later, as Ten-m mutants failed to display a pair-rule phenotype, a different role was proposed and Ten-m/Odz genes were demonstrated to code for transmembrane proteins with a possible oscillator function [9,10,11]. In mice and humans, four different Ten-m paralogous genes (Ten-m1–4) code for large type II transmembrane proteins, called teneurin (TENM)1–4.

A comparison analysis of teneurins expression in vertebrates and invertebrates shows a highly conserved expression pattern in different tissues during development [12]. Teneurins are mainly expressed in the development of the central nervous system. A different but partially overlapping loco-regional tissue distribution of teneurins has been demonstrated [1,13]. Indeed, high levels of TENM2, TENM3 and TENM4 have been found in the caudal regions of the cerebral cortex, while TENM1 expression is confined in the more rostral areas [1,13,14,15,16]. Besides the well-documented topographic distribution of teneurins and their role in neuronal development, recent evidence from in vivo experiments using teneurin knock out (KO) mouse models has indicated that these proteins may play an important role in determining the patterns of neural circuitry.

## 2. Common Structural and Functional Features of Mammalian Teneurins

**Figure 1:**
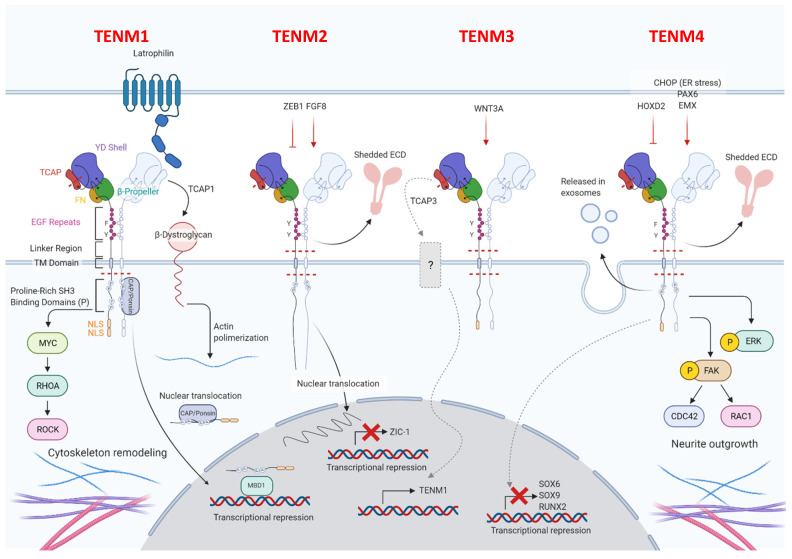
Schematic representation of teneurins’ structure and their molecular interactions. Teneurins display different major domains: the C-terminal extracellular domain, the transmembrane domain (TM domain) and the N-terminal intracellular domain. The C-terminal domain is composed of a globular part divided into a teneurin C-terminal associated peptide (TCAP), a tyrosine-aspartic acid (YD)-repeat barrel, a 6-bladed β-propeller domain, and a fibronectin plug (FN) domain. The globular portion of the protein is responsible for teneurins’ protein–protein interactions with, for example, latrophilins. The globular part of the protein is followed by eight epidermal growth factor (EGF) modules, that differ among teneurins. Indeed, while TENM1 and TENM4 have a phenylaniline (F) in the third residue of the fifth EGF repeat, TENM2 and TENM3 possess, instead, a tyrosine residue (Y). Teneurins cross the membrane via some hydrophobic residues that are linked to the N-terminal intracellular domain. Among the transmembrane domain and the EGF repeats, TENM2–4 have a cleavage site (represented by the red dashed lines) that allows the release of the extracellular domain (ECD). Finally, all teneurins possess a cleavage site in the intracellular domain allowing the intracellular portion to be processed and translocated to the nucleus and a cleavage that allows the release of TCAP. The N-terminal domain is characterized by one or more proline-rich SH3-binding domains, four in TENM1 and two in TENM2 and TENM4, and it terminates with predicted nuclear localization sequences (NLS), two in TENM1 and one in TENM3 and TENM4. This graphical representation also shows the functional interactions between teneurins and other proteins. The cytoskeleton remodeling operated through TENM1 action via a Myc-dependent transcriptional up-regulation of ras homolog family member A (RhoA) and consequent rho-associated protein kinase (ROCK) activation is shown. Moreover, TENM1 binds CAP/ponsin, which translocate in the nucleus. In the nucleus, the intracellular domain of TENM1 is able to bind MBD1, a DNA binding protein acting as transcription repressor. Moreover, TCAP1 can be released and bind β-distroglican inducing actin polymerization. TENM2 expression can be induced by fibroblast growth factor 8 (FGF8) and inhibited by Zinc finger E-box binding homeobox (ZEB1). Moreover, TENM2 nuclear translocation inhibits zic-1 expression. TENM3 expression is induced by Wnt3a, and TCAP3 release is able to impair TENM1 expression. TENM4 expression is induced by CAAT enhancer binding protein homologous protein (CHOP), upon endoplasmic reticulum stress, PAX6 and EMX, while it is inhibited by HOXD2. Moreover, TENM4 increases ERK phosphorylation as well as focal adhesion kinase (FAK), which causes the activation of CDC42 and RAC1, inducing neurite outgrowth. Differently, TENM4 higher expression decreases SOX6, SOX9 and RUNX2 expression. “Created with BioRender.com”.

All four of the identified mammalian teneurins are type II transmembrane proteins and possess strong sequence homology, consist of about 2800 amino acids, and entail a complex set of functional domains. Although their involvement in cell adhesion has been well documented, teneurins are unusual in that they lack the classical domains that are generally observed in cell adhesion molecules, such as immunoglobulin, cadherin, laminin-a, neurexin and sex hormone-binding globulin, and integrin domains. All teneurins have a C-terminal extracellular domain, including a globular part, followed by an Ig-like domain (composed of a carboxypeptidase-like domain and of a cysteine-rich domain), which is involved in teneurins dimerization, and by eight EGF modules (Figure 1).

The globular part of all teneurins is composed of a fibronectin plug, a protein–protein interaction domain, called 6-bladed β-propeller, a tyrosine-aspartic acid (YD)-repeat barrel, an RHS core protein domain and a teneurin C-terminal associated peptide (TCAP), which is involved in the interaction between teneurins and latrophilins [17]. TCAP has recently been demonstrated to also possess nuclease activity and an apoptogenic role [18]. Teneurin proteins cross the membrane via hydrophobic residues that are linked to an N-terminal intracellular domain, which is characterized by one or more proline-rich SRC Homology 3 (SH3)-binding domains in the part closest to the transmembrane region, conserved tyrosines, which have been predicted to be phosphorylated, nuclear localization sequences, and a basic sequence motive RKRK, which may be a proteolytic cleavage site [19]. Despite the great homology, some structural differences between teneurins exist, and these are mainly related to the number of proline-rich SH3-binding domains, and also to the presence or absence of predicted nuclear localization sequences and proteolytic cleavage sites [19]. Details on the distinguishing features of each teneurin will be described in the following dedicated paragraphs.

Teneurins, following dimerization, act as transcriptional regulators after the release of the intracellular domain. They can form both homo- and heterodimers. Electrophoretic and electron-microscopic analyses of TENM1 have shown that homodimerization most likely takes place between the EGF-like modules and the hydrophobic regions of two teneurins that are arrayed side-by-side on the same cell [20]. Recent evidence has revealed that the homodimerization can also occur between two identical teneurins expressed on different cells in close proximity [21]. This trans-homodimerization of teneurins has been reported to allow the correct matching between axons and their targets to occur, thus contributing to correct circuit-wiring in the nervous system [22,23,24]. Upon homodimerization, teneurins’ intracellular domains are cleaved close to the plasma membrane and translocate into the nucleus, where they regulate gene expression via direct and indirect interactions with transcription factors [25].

In addition, all teneurins can mediate cell–cell interaction by forming heterodimers [26] with different proteins, among which are the adhesion G-protein-coupled receptors latrophilins and the actin-crosslinking protein filamin. While the trans-heterodimerization between teneurins and latrophilins is involved in synapse formation and organization [27,28] and in cellular communication [29], teneurins and filamin interactions are involved in cell motility [9]. Recent X-ray crystallographic studies have revealed that the teneurin–latrophilin binding involves the presence of another protein, the fibronectin leucine-rich repeat transmembrane protein (FLRT) [30].

Teneurins exist as splice variants which differ for the presence or absence of nine- and seven-amino acid insertions at the EGF repeats and the β-propeller regions, respectively [21,31,32]. The different teneurins’ alternative variants interact with different ligands, mediating either teneurin homodimerization or heterodimerization with latrophilins, leading to the activation of different biological functions [21]. This, together with the possibility that they can be cleaved and released, is likely to contribute to the high level of complexity in these molecules and their functions. Indeed, amino acid sequence analyses have shown that teneurin proteins possess cleavage sites in the extracellular domain, leading to potential for the extracellular release of teneurins. One cleavage site can be located between the transmembrane and EGF-like domains, and is susceptible to proteolytic cleavage in vitro [20], while the other is situated near the C-terminus of the protein [33] and is responsible for the secretion of the TCAP domain, which is a bioactive neuropeptide involved in several functions.

Besides their wide expression in developing embryos, all teneurins can be found in several adult tissues, suggesting that they have a potential role in normal physiology. However, both their function and whether their expression is essential in normal adult tissues are currently unknown.

An ever-growing amount of evidence has demonstrated the aberrant expression [34] as well as somatic alterations and chromosomal rearrangements [35] of teneurins in human tumors of different histotypes. Their involvement in tumor cell signaling, tumorigenic regulatory pathways and drug resistance mechanisms have also been described [34]. Moreover, thanks to an analysis of open-access molecular and clinical datasets of tumor patients, the possible prognostic impact of teneurins has also been reported [35]. However, their functional role in tumor initiation and malignant progression has not yet been fully elucidated. Herein, we review available data about the general features of teneurins with a particular focus on their involvement in cancer, in order to highlight the potential role that teneurins may be able to play as novel tumor biomarkers and targets for therapeutic approaches.

## 3. TENM1

TENM1 is initially expressed in the nervous system during embryogenesis, thus suggesting that it has a physiological role in neural development. Specifically, it is expressed in the cerebral cortex, in mice, but also in the midbrain, spinal cord, hind brain and in the trigeminal ganglion. A weak expression has also been observed in the olfactory epithelium and in the olfactory bulbs [14]. TENM1 expression is conserved in adult mouse tissues, including the cortex, thalamus, hippocampus, and the granular layer of the cerebellum [14]. Recent data have demonstrated a role for TENM1 in the establishment of olfactory circuits; indeed, TENM1 deletion in mice affects their ability to detect odors and TENM1 mutations in humans are correlated with congenital anosmia [36].

TENM1 has some peculiar structural features that distinguish it from the other teneurins (Figure 1). In particular, two nuclear localization sequences and four SH3 binding domains are present in the intracellular domain of the protein [19]. Moreover, as has also been observed for TENM4, a phenylalanine moiety is present in the third residue of the fifth EGF repeat [19], and this has been demonstrated to be essential for teneurins’ dimerization [20]. Furthermore, it should be noted that TENM1 lacks the predicted furin cleavage site between the transmembrane domain and the EGF modules, present in other members of the family [19].

As far as signaling is concerned, the TENM1 intracellular domain can be cleaved, upon homodimerization, and enter into the nucleus. However, the exact mechanism of TENM1 cleavage is still not fully understood [4]. Two different proteins that interact with the TENM1 intracellular domain have been identified. One is the methyl-CpG-binding domain protein 1 (MBD1), a DNA-binding protein acting as transcription repressor, and the other is the c-Cbl-associated protein CAP/ponsin, which is a widely expressed adaptor protein that contains SH3 domains. While the biological significance of the interaction of the TENM1 intracellular domain with MBD1 is unknown, its binding with CAP/ponsin is involved in cell adhesion, in cytoskeleton remodeling [4], and in growth factor-mediated signaling [37].

A specific TENM1 TCAP (TCAP-1) has been identified, both in vitro and in vivo, thanks to the presence of the furin cleavage site at the C-terminal domain. Recent data have demonstrated that TCAP-1 may either derive from full-length pro-protein cleavage, or may be released via the transcription of an independent mRNA that specifically encodes the last exon of the TENM1 TCAP region [38]. TCAP1 has potent bioactivity, as it is involved in neuronal cells’ protection against stress- and hypoxia-induced cell death [39], upon beta-dystroglican binding and consequent mitogen-activated protein kinase (MEK) extracellular signal-regulated kinase (ERK) signaling activation [40,41], as well as in neuroplasticity [42]. It has recently been suggested that all of these effects depend upon the TCAP-1-mediated inhibition of corticotropin-releasing factor (CRF) activity, which leads to increased energy production [38]. Indeed, in vivo studies in rodents have demonstrated that TCAP-1 administration impacts upon CRF-associated behavior, such as anxiety and depression, and increases glucose levels in rat brains [38].

### TENM1 and Cancer

**Figure 2:**
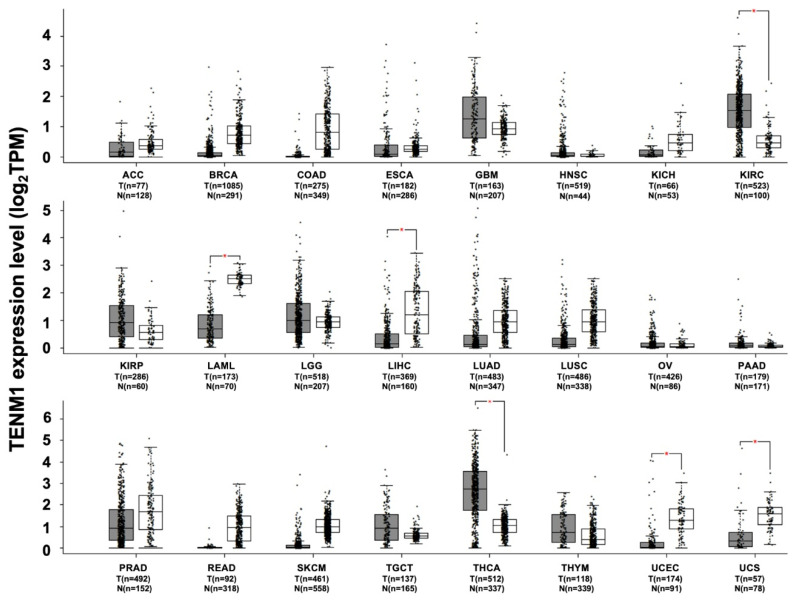
TENM1 expression in different datasets of tumors and normal samples. TENM1 expression level, shown as log2 of transcript per million mapped reads value, was analyzed from RNA sequencing data of tumors and normal samples from the TCGA and the GTEx projects, using a web standard processing pipeline [43]. Tumor (T) and normal samples (N) are represented in grey and white box plots, respectively, each dot representing a sample (*n* = number of samples within the dataset). Statistical differential analysis was performed using the Gene Expression Profiling Interactive Analysis (GEPIA) default recommended parameters, one-way ANOVA, |Log2FC| ≥ 1 and *p*-value ≤ 0.01. Datasets description: ACC; Adrenocortical carcinoma. BRCA; Breast invasive carcinoma. COAD; Colon adenocarcinoma. ESCA; Esophageal carcinoma. GBM; Glioblastoma multiforme. HNSC; Head and neck squamous cell carcinoma. KICH; Kidney chromophobe. KIRC; Kidney renal clear cell carcinoma. KIRP; Kidney renal papillary cell carcinoma. LAML; Acute myeloid leukemia. LGG; Brain lower grade glioma. LIHC; Liver hepatocellular carcinoma. LUAD; Lung adenocarcinoma. LUSC; Lung squamous cell carcinoma. OV; Ovarian serous cystadenocarcinoma. PAAD; Pancreatic adenocarcinoma. PRAD; Prostate adenocarcinoma. READ; Rectum adenocarcinoma. SKCM; Skin cutaneous melanoma. TGCT; Testicular germ cell tumors. THCA; Thyroid carcinoma. THYM; Thymoma. UCEC; Uterine corpus endometrial carcinoma. UCS; Uterine carcinosarcoma.

Besides congenital general anosmia, TENM1 deregulation has also been associated with several tumors. By exploiting the web-based tool Gene Expression Profiling Interactive Analysis, or GEPIA (http://gepia.cancer-pku.cn, access date 28 January 2021), to analyze RNA sequencing data from TCGA and GTEx projects, a significant increase in TENM1 expression has been found in thyroid carcinoma and in kidney renal clear cell carcinoma datasets compared to paired normal samples. A trend of increase has also been observed in the glioblastoma dataset. By contrast, in acute myeloid leukemia, liver hepatocellular carcinoma, uterine corpus endometrial carcinoma and uterine carcinosarcoma, a significant decrease in TENM1 expression is found compared to normal tissue samples (Figure 2). However, data on the association between TENM1 deregulation and tumor progression are scarce and confined to a few tumor types, such as thyroid carcinoma, pituitary tumor and glioblastoma.

Further support for TENM1′s functional contribution to carcinogenesis, TENM1 mutations and chromosomal alterations have occasionally been found in tumors of differing origins. For instance, TENM1 is one of the most mutated genes in melanoma [44], and is frequently mutated in B-cell tumors [45], BRCA-mutated luminal breast cancer [46] and familiar low-grade glioma [47]. In the case of thymoma [48], prostate and kidney adenocarcinomas and acute myeloblastic and myeloid leukemia [49,50], cytogenetic rearrangements involving TENM1 have been demonstrated. Moreover, even if not univocal, a correlation between TENM1 expression in different cancers and patients’ survival outcome can be observed [35].

A gene expression study, performed on normal and papillary thyroid carcinoma tissues, identified TENM1 as one of the overexpressed genes in tumor, as compared to normal, tissues. This indicates that there is a potential oncogenic role of TENM1 in this tumor histotype [51]. Moreover, by comparing gene expression profiles during papillary thyroid carcinoma progression, Cheng and colleagues have found a correlation between the increase in TENM1 expression and disease progression from stage I to stage IV [52]. The increasing expression of TENM1 from the less to the more advanced tumor stages suggests its involvement in tumor progression and invasion, and pinpoints its potential role, not only as an oncogene, but also as a diagnostic biomarker. The possible oncogenic role of TENM1 in thyroid cancer progression is also supported by the fact that bioinformatics analyses and luciferase reporter assays have recently identified TENM1 as a direct functional target for miR-486 in this type of tumor. Normally, miR-486 targets TENM1, decreasing its expression [53]. The miR-486 down-regulation frequently observed in papillary thyroid carcinoma cells should thus justify the increased expression of TENM1. Interestingly, a statistically significant correlation between miR-486 down-regulation and worse overall survival was also observed in patients. Moreover, miR-486 restoration in papillary thyroid carcinoma cells significantly inhibited tumor cell proliferation, migration and invasion in vitro, via ERK and protein kinase B (Akt) signaling pathways, as well as inhibiting epithelial-to-mesenchymal transition (EMT) regulation and tumor cell growth in vivo [53]. Recent data show that TENM1 is also up-regulated in a follicular variant of papillary thyroid cancer [54], and it is significantly more highly expressed and mutated in thyroid malignant nodules than in benign ones [55]. In line with these findings, the up-regulation of TENM1 mRNA was found in invasive and aggressive-invasive prolactin pituitary tumors as compared to non-invasive ones, suggesting its association with tumor progression also in this tumor histotype [56].

Another tumor in which TENM1 seems to play an oncogenic role is glioblastoma, where it promotes the cell proliferation, the cytoskeletal remodeling of tumor cells and the invasion of the surrounding environment, both in vitro and in vivo, via the Myc-dependent transcriptional up-regulation of ras homolog family member A (RhoA) and consequent rho-associated protein kinase (ROCK) activation. Indeed, the absence of TENM1, achieved via gene deletion or down-regulation by small interfering RNA (siRNA), drastically reduces the invasive capacity of glioblastoma cells. Moreover, reduced survival has been found in mice challenged with TENM1-overexpressing glioblastoma cells, and an inverse correlation between TENM1-positive cells and overall and progression-free survival has been found in samples derived from glioblastoma patients [57]. Interestingly, hypoxia has been identified as an extracellular activator of TENM1 expression in glioblastoma. Indeed, TENM1 has been observed to be mainly concentrated in highly hypoxic tumor regions in surgical specimens from patients affected by glioblastoma [58]. Moreover, TENM1 mRNA up-regulation in response to hypoxia increases cell migration capacity in glioblastoma cells, while the blockade of TENM1 expression results in reduces hypoxia-induced glioblastoma cell migration [58]. Consequently, TENM1-targeting could be proposed as a new strategy for glioblastoma therapy, as TENM1 overexpression is induced by hypoxia, and a hypoxic tumor microenvironment is associated with treatment resistance and poor prognosis.

TENM1 has been implicated in stemness and cancer cell differentiation both in glioblastoma and prostate cancer. Indeed, it has been demonstrated that TENM1 expression enables glioblastoma-derived cells to form neurospheres and to invade the surrounding microenvironment, while TENM1 down-regulation, through specific short hairpin RNA (shRNA), drastically halts glioblastoma cells’ invasive capacity. Interestingly, TENM1-positive cells are those localized at the periphery of the neurospheres and are those endowed with higher migratory capacity. Similar results have also been described in prostate adenocarcinoma, where higher TENM1 expression is found in undifferentiated cells that display a higher tumorsphere-forming ability compared to more differentiated cells [59].

Finally, although available data on the role of TENM1 in the metastatic processes are scarce, its involvement should not be excluded. Indeed, a proteomic analysis of myxoid liposarcoma biopsies has shown an inverse correlation between the level of TENM1 expression and metastasization [60]. Interestingly, a single-cell analysis of circulating tumor cells, performed on a lung cancer patient, highlighted the presence of an acquired TENM1 single nucleotide variation in circulating tumor cells, whose function has not been elucidated. The same mutation was present in the patient’s liver metastasis [61].

## 4. TENM2

TENM2, as the other components in the teneurin family, is expressed in the central nervous system during embryonic development, particularly in the forebrain, rostral and central midbrain [14]. It is highly expressed in the visual cortex [13,62] where it is involved in binocular visual circuits, as demonstrated in experiments performed using TENM2 KO mice [63]. In humans, TENM2 has also been detected in odontoblasts and limbs, with a role in cell differentiation during development, and in adipocyte precursor cells where it maintains the white adipocyte phenotype [64,65]. It is interesting to note that in chicken limb development, TENM2 expression at the apical ectodermal ridge has a similar pattern of expression as fibroblast growth factor 8 (FGF8). Indeed, the exogenous administration of FGF8 induces TENM2 expression [32]. In adult mice, TENM2 is also expressed in organs other than the nervous system, such as the kidney and testes [12].

Now turning to the distinctive features of TENM2 (Figure 1), it should be noted that this protein has a furin cleavage site just outside the plasma membrane, in the region between the transmembrane domain and the EGF-like repeats [32]. The presence of this site allows a soluble portion of the protein to be released into the extracellular milieu [2]. Moreover, TENM2 possesses a tyrosine in the third residue of the fifth EGF repeat, and the intracellular region has two SH3-binding domains [19]. It has been shown that the intracellular domain of TENM2, cleaved and released after TENM2 dimerization, translocate into the nucleus, where it represses Zic-1 mediated transcription, promoting cellular differentiation [3].

Interestingly, in vitro experiments of artificial synapse formation have shown that the two TENM2 splice variants regulate the capability of the protein to induce the formation of either inhibitory or excitatory synapsis [21]. In particular, the trans-homodimerization of the longer variant is important for inhibitory synapse induction [31], while the FLRT3-mediated binding with latrophilin regulates the formation of excitatory synapses [21].

### TENM2 and Cancer

**Figure 3:**
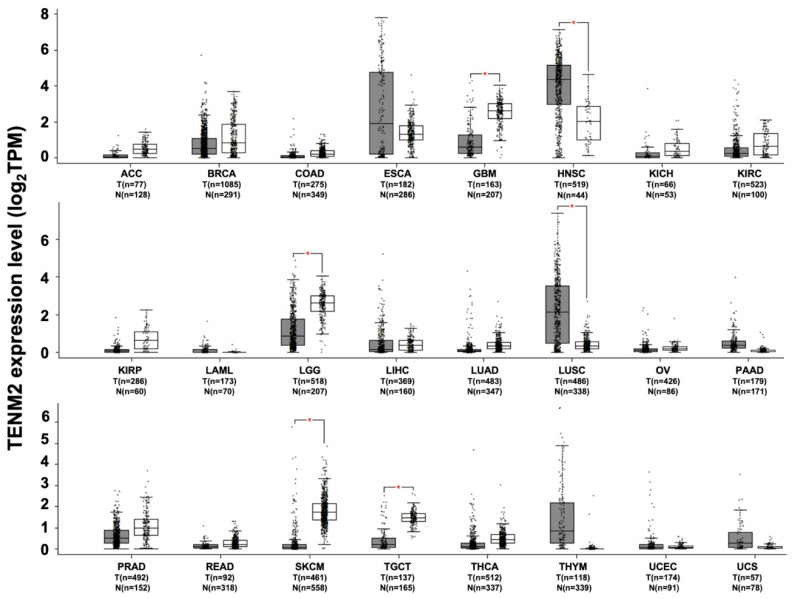
TENM2 expression in different datasets of tumors and normal samples. TENM2 expression level, shown as log2 of transcript per million mapped reads value, was analyzed from RNA sequencing data of tumors and normal samples from the TCGA and the GTEx projects, using a web standard processing pipeline [43]. Tumor (T) and normal samples (N) are represented in grey and white box plots, respectively, each dot representing a sample (*n* = number of samples within the dataset). Statistical differential analysis was performed using the GEPIA default recommended parameters, one-way ANOVA, |Log2FC| ≥1 and *p* ≤ 0.01. Datasets description: ACC; Adrenocortical carcinoma. BRCA; Breast invasive carcinoma. COAD; Colon adenocarcinoma. ESCA; Esophageal carcinoma. GBM; Glioblastoma multiforme. HNSC; Head and neck squamous cell carcinoma. KICH; Kidney chromophobe. KIRC; Kidney renal clear cell carcinoma. KIRP; Kidney renal papillary cell carcinoma. LAML; Acute myeloid leukemia. LGG; Brain lower grade glioma. LIHC; Liver hepatocellular carcinoma. LUAD; Lung adenocarcinoma. LUSC; Lung squamous cell carcinoma. OV; Ovarian serous cystadenocarcinoma. PAAD; Pancreatic adenocarcinoma. PRAD; Prostate adenocarcinoma. READ; Rectum adenocarcinoma. SKCM; Skin cutaneous melanoma. TGCT; Testicular germ cell tumors. THCA; Thyroid carcinoma. THYM; Thymoma. UCEC; Uterine corpus endometrial carcinoma. UCS; Uterine carcinosarcoma.

TENM2 mRNA down-regulation, compared to normal tissues, has been observed in brain lower grade glioma, in skin cutaneous melanoma, in testicular germ cell tumors (Figure 3), in cervical intraepithelial neoplasia [66] and in esophagus squamous cell carcinoma [67]. Moreover, while the TENM2 protein has been found to be highly expressed in normal squamous cells of the stomach and small intestine, in the majority of stomach tumors, the level of expression of TENM2 protein was either low or undetectable [68]. The possible tumor suppressor role of TENM2 is also suggested by the observation that hepatitis B virus-related insertional mutagenesis, leading to TENM2 gene disruption, is frequently associated with hepatocarcinogenesis [69].

In a large-scale genomic and proteomic analysis of progressive stages of prostate cancer [70], a gradual reduction in TENM2 was observed, passing from clinically locally advanced to metastatic cancer. Similarly, a comparison of the gene-expression profiles of tumors from early-stage cervical cancer patients, with or without lymph node metastases, has revealed that TENM2 is one of the genes that undergo down-regulated expression [71]. Furthermore, data from a microarray of micro-dissected epithelial cells have displayed significantly reduced TENM2 expression in breast hyperplastic enlarged lobular units, which are histologically considered premalignant precursors of breast cancer, compared to normal terminal duct lobular units [72]. In the case of ovarian cancer, the analysis of a cohort of serous tumors has revealed that TENM2 expression is significantly lower in poorly differentiated (grade III) and undifferentiated tumors, compared to well differentiated (grade I and II) tumors. As loss of differentiation is a typical feature of increased malignancy and aggressiveness, these data indicate that TENM2 may play an oncosuppressor role [73]. Interestingly, despite the presence of predicted CpG islands in introns from TENM2 genomic regions, the in vitro treatment of ovarian cancer cells with demethylating agents failed to induce the expression of TENM2, suggesting that, at least in ovarian cancer cells, TENM2 down-regulation in aggressive tumors is not related to epigenetic factors. Indeed, in vitro experiments using ovarian cancer cell lines have demonstrated that TENM2 expression is induced in response to the oncogenic growth factor FGF8, and that the control of TENM2 gene expression is cell type-dependent, and might relay on the expression of a particular FGF receptor isoform [73]. Similarly, TENM2 down-regulation has been observed in breast cancer cells that were concomitantly treated with prolactin and 17β-estradiol, stressing the fact that its down-regulation is not related to epigenetic factors [74].

Gene-based expression-profile analyses using the Human Protein Atlas data sets (https://www.proteinatlas.org/pathology) suggest that TENM2 expression has potential relevance as a prognostic marker in a range of tumors. In colorectal, pancreatic, prostate and ovarian cancers, low levels of TENM2 expression are correlated with lower patients’ overall survival [35]. A conflicting trend can be observed in urothelial, endometrial, head and neck, renal, stomach and thyroid cancers, as well as in glioma and melanoma, in which low levels of TENM2 expression are correlated with better patients’ overall survival [35]. Moreover, TENM2 up-regulation in tumors as compared to normal tissues is found in head and neck squamous cell carcinoma, lung squamous cell carcinoma and thymoma (Figure 3). In line with this in silico data, a proteomic analysis of lung-tumor samples has identified TENM2 expression as a marker with which to better classify neoplastic lesions in the lung, as it is highly and homogenously expressed in pleural mesothelioma compared to lung adenocarcinoma cell lines [68]. Accordingly, an analysis of triple negative breast cancer (TNBC) patient samples has suggested that there is a significant correlation between high TENM2 expression and reduced patient metastatic-free survival time [75]. Interestingly, in TNBC cell lines, TENM2 seems to be regulated by the Zinc finger E-box binding homeobox 1 (ZEB1), a transcription regulator with a potential role in cancer progression that is involved in promoting the EMT. Indeed, transcriptional analysis of wild type and ZEB1 KO cells revealed TENM2 as one of the up-regulated genes in ZEB1-deficient cells [75].

A possible link between TENM2 deregulation and the drug sensitivity of cancer cells has also been proposed, again with contradictory results. While ovarian cancer cells resistant to vincristine have higher TENM2 expression levels compared to parental cells [76], their cisplatin sensitivity is decreased upon TENM2 down-regulation [73]. The overexpression of TENM2 has also been observed in Erlotinib-resistant lung adenocarcinoma cells, compared to parental cells [77].

As observed for TENM1, the TENM2 gene is frequently altered in tumors, further hinting at its involvement in cancer development and progression. Cytogenetic rearrangements involving the TENM2 gene have indeed been observed in neuroblastoma [78,79], prostate adenocarcinoma [50], invasive breast carcinoma [50,80], lung adenocarcinoma [49,50,78] and breast and ovarian serous adenocarcinoma [49,50].

While all the results described so far focus on the role of TENM2 deregulation in tumor cells, it is worth noting that TENM2 can also be found in the cells of the microenvironment. Indeed, in a gene expression profiling study performed on normal and cancer-associated fibroblasts derived from breast cancer patients, TENM2 mRNA was one of the large number of genes that were found to be up-regulated [81]. Indeed, cancer-associated fibroblasts play an important role in the tumor microenvironment, promoting angiogenesis and secreting cytokines that increase cell growth and thus support tumor development. Further evidence supporting a possible role for TENM2 deregulation in the tumor microenvironment comes from whole-genome single nucleotide polymorphism profiling, which compared the progressive passages of tumor-derived endothelial cells. Parallel to the acquisition of the higher-level expression of endothelial-specific genes and proteins, and greater endothelial-like behavior in vitro, late-passage tumor-derived endothelial cells acquired interstitial chromosomal gains and losses that affected a relatively small number of genes, including TENM2 [82].

## 5. TENM3

TENM3, like the other teneurins, has a pivotal role in the development of the central nervous system. In situ hybridization and tracing studies, using wild type and TENM3 KO mice, have revealed its importance for the generation of the pattern of connectivity in the hippocampus, and in the visual and striatal system [83]. In the hippocampus, normal TENM3 expression is required to generate the correct circuitry between neurons from the CA1 region and those from the distal subiculum [31]. During development, TENM3 becomes particularly highly expressed in the visual cortex and in the retina, showing, in both tissues, a high ventral and low dorsal gradient of expression [62]. It has been proposed that these topographically corresponding gradients of protein expression are responsible for the establishment of precise matching between afferent axons and target cells, which would suggest that TENM3 plays a potential role as a chemoaffinity molecule [83]. Proof of the importance of TENM3 in visual system circuit connectivity has been found in behavioral studies that show that TENM3 KO mice lack binocular vision [62]. In the interconnected regions of the thalamus, TENM3 expression shows a dorsal-to-ventral gradient [15], hinting that the protein may also have a key role as a chemoaffinity molecule in this region of the central nervous system. Indeed, anterograde tracer studies performed using wild type and TENM3 KO mice have revealed the presence of abnormal thalamus circuitry, which delayed the acquisition of motor skills [15], orientation selectivity and the ability to recognize shape and position stimuli in mice [84]. Besides the central nervous system, TENM3 is also expressed during the development of limbs, the urogenital system and the mesentery of the gut [14,85]. Moreover, in mice, its expression is also conserved in a few adult tissues, including the brain, liver and testes [20,85]. Recently, TENM3 expression has been shown to be regulated by Wnt signaling. Indeed, a significant increase in TENM3 mRNA and protein expression levels was observed when exogenous Wnt3a, a canonical Wnt ligand, is administered to neuroblastoma cells [86] and human colon myofibroblasts [87].

As far as TENM3 structure is concerned (Figure 1), two distinctive features, related to the intracellular domain, can be found: it lacks the SH3-binding domain and only has one nuclear localization sequence [88]. In the extracellular domain, a predicted furin cleavage site is present between the transmembrane domain and the EGF modules. Moreover, different TENM3 splice variants exist and can influence protein–protein interactions. In particular, all splice variants, except one, allow homodimerization to occur between two TENM3, while heterodimerizations between TENM3 and latrophilin3 are allowed by a single splice variant only [28,31]. A TCAP of TENM3 (TCAP-3) has been identified. Although the specific roles of TCAP-3 need to be better elucidated, evidence in rats suggests that it potentially plays a role in reproduction and neuron proliferation. Indeed, in rats, TCAP-3 is expressed in the testes, where it influences testosterone production [89], while in neuronal cells, a synthetic TCAP-3 has been shown to stimulate cAMP production, probably by binding a G-coupled receptor [33].

### TENM3 and Cancer

**Figure 4:**
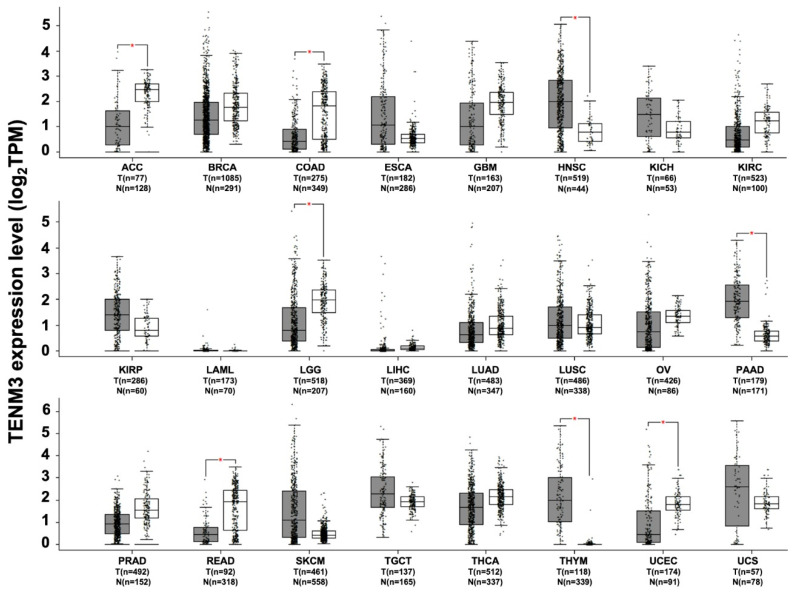
TENM3 expression in different datasets of tumors and normal samples. TENM3 expression level, shown as log2 of transcript per million mapped reads value, was analyzed from RNA sequencing data of tumors and normal samples from the TCGA and the GTEx projects, using a web standard processing pipeline [43]. Tumor (T) and normal samples (N) are represented in grey and white box plots, respectively, each dot representing a sample (*n* = number of sample within the dataset). Statistical differential analysis was performed using the GEPIA default recommended parameters, one-way ANOVA, |Log2FC| ≥1 and *p* ≤ 0.01. Datasets description: ACC; Adrenocortical carcinoma. BRCA; Breast invasive carcinoma. COAD; Colon adenocarcinoma. ESCA; Esophageal carcinoma. GBM; Glioblastoma multiforme. HNSC; Head and neck squamous cell carcinoma. KICH; Kidney chromophobe. KIRC; Kidney renal clear cell carcinoma. KIRP; Kidney renal papillary cell carcinoma. LAML; Acute myeloid leukemia. LGG; Brain lower grade glioma. LIHC; Liver hepatocellular carcinoma. LUAD; Lung adenocarcinoma. LUSC; Lung squamous cell carcinoma. OV; Ovarian serous cystadenocarcinoma. PAAD; Pancreatic adenocarcinoma. PRAD; Prostate adenocarcinoma. READ; Rectum adenocarcinoma. SKCM; Skin cutaneous melanoma. TGCT; Testicular germ cell tumors. THCA; Thyroid carcinoma. THYM; Thymoma. UCEC; Uterine corpus endometrial carcinoma. UCS; Uterine carcinosarcoma.

Evidence of its deregulation in different types of cancer has suggested that TENM3 may have a possible functional role in tumorigenesis. The in silico analysis of RNA sequencing data from the TCGA and GTEx projects demonstrates that TENM3 is significantly more highly expressed, as compared to normal tissues, in head and neck squamous cell carcinoma, in pancreatic adenocarcinoma, in thymoma (Figure 4), and in neuroblastoma [90]. High TENM3 expression is observed in the human neuroblastoma cell line SH-SY5Y, in which TENM3 is one of the most abundant membrane proteins [91]. Moreover, TENM3 was found to be up-regulated in the mammary glands of nulliparous women [92], who have a higher incidence of breast cancer than parous ones. Due to the higher number of stem cells that do not complete the differentiation process, which is normally accomplished during pregnancy [93], TENM3 up-regulation in the undifferentiated cells of the mammary glands may be related to malignant transformation [92]. The treatment of breast cancer cell lines with indole-3-carbinol, an anti-tumoral agent, is able to down-regulate TENM3 expression, which supports the possible role of TENM3 overexpression in tumor development [94].

Data from different tumor types has suggested that TENM3 may possibly contribute to cancer metastasization. Indeed, TENM3 is overexpressed in a selected MDA-MB-231 cell population with increased cell migration ability [95]. Moreover, TENM3 staining increased in bone metastasis, compared to primary prostate tumors [96], and is up-regulated in colorectal cancer, with lymphovascular and perineural cancer cell invasion. Increased TENM3 copy numbers and expressions have also been found in glioblastoma patients with leptomeningeal dissemination, compared to patients who do not present this pattern of metastasization [97]. The possible pro-metastatic role of TENM3 can also be hypothesized in lung tumors in which patients’ circulating tumor cells display TENM3 mutations that are also maintained in metastasis, suggesting that these mutations are important for the migration process [61]. Interestingly, a gene-based query at the Human Protein Atlas displayed a correlation between worst survival and high TENM3 expression in most of the tumors analyzed, including endometrial, lung, ovarian, stomach, thyroid, urothelial cancer and glioma [35].

Although the majority of the available data hint at an oncogenic role of TENM3 in cancers, an oncosuppresor role cannot be excluded. Indeed, TENM3 down-regulation, compared to normal tissues, has been observed in several tumors, including brain lower grade glioma, adrenocortical, colon, rectum, and uterine corpus endometrial carcinomas (Figure 4). In silico analyses of data from the Human Protein Atlas show that TENM3 mRNA down-regulation is correlated with poor patient prognosis in cervical, pancreatic and renal cancers [35]. Moreover, in a whole-genome sequence analysis of about 90 neuroblastomas of all stages, a correlation between TENM3 mRNA deregulation and poor patients’ prognosis has been observed [98]. Interestingly, in this study, TENM3 was identified as one of the most frequently altered genes, while the chromothripsis that affected the TENM3 gene was found in about 20% of high-risk neuroblastoma. The TENM3 gene has been also identified as a frequent integration site for human papilloma virus DNA in cervix cancer cells [99], and its hemizygous deletion has been observed in glioblastomas [100]. Although no translocations have been reported for TENM3, a high frequency of TENM3 mutation has been found in skin cutaneous melanoma and pancreatic adenocarcinoma [101,102], suggesting that TENM3 may also play a role in carcinogenesis in these tumor types, and in others that have not yet been investigated.

Recently, for the first time, the possibility of using teneurins as potential suitable targets for anti-tumor immune therapy has been demonstrated. TENM3 has been identified as one of the neoantigens expressed by ovarian cancer in recurrent patients. Vaccination of these patients with autologous dendritic cells pulsed with autologous whole-tumor cell lysate resulted in an effective anti-tumor immune response that correlated with increased patient survival. Interestingly, a specific TENM3-mutated epitope CD8^+^ T cell response was observed when peripheral blood mononuclear cells were re-stimulated [103].

## 6. TENM4

TENM4 mRNA has been found in the hippocampus, in the trigeminal ganglia and the cerebral cortex of developing mouse brains. Herein, TENM4 is involved in the regulation of cortical area patterning [13], and it is normally expressed by cortical neurons and their precursors in a high caudal to low rostral gradient that matches that of the homeobox transcriptional regulator EMX2. The significant reduction in TENM4 mRNA and the loss of the expression gradient observed in EMX2 KO mice suggest that TENM4 expression is regulated by EMX2 [13]. TENM4 is also expressed in non-neuronal developing tissues of mesodermal derivation, such as the mesentery, and forming limb and bone, but also the trachea, adipose tissue and skin [12]. Interestingly, in developing mouse limbs, the depletion of the homeobox D (HoxD) transcription factor involved in morphogenesis is associated with increased TENM4 expression, which suggests that TENM4 is a possible HoxD target [104]. TENM4 expression is regulated also by Emx2 and PAX6 proteins [13]. In adult mice, besides the brain, a low expression of TENM4 mRNA has also been observed in the liver, testes and thymus [12,20], while in humans TENM4 has been detected in the female gonads [73].

Although the overall domain organization of TENM4 is comparable to that of the other teneurins, some distinctive features can be recognized (Figure 1). The TENM4 protein bears a phenylalanine in the third residue of the fifth EGF repeat in the extracellular domain, and, in the intracellular domain, two SH3-binding domains and one nuclear localization sequence. Lastly, TENM4 lacks the predicted furin cleavage sequence just outside the plasma membrane [19].

Considerable amounts of data indicate the involvement of TENM4 in neuronal and chondrocyte cell differentiation. TENM4 is expressed in differentiated neurons where it plays a key role in regulating neurite outgrowth, through focal adhesion kinase (FAK) signaling. Indeed, TENM4 KO neuroblastoma cells of mouse origin display decreased neurite length and ability to generate filopodia-like protrusions through FAK, Cdc42 and Rac1, whereas TENM4 overexpression in neuroblastoma cells promotes protrusion formation [105]. Oligodendrocytes are another cell lineage in which TENM4 acts as a regulator of differentiation. Here, its disruption limits the generation of normal oligodendrocyte processes, leading to lower axon myelination, which results in the essential tremor phenotype in mice [106]. Moreover, a similar phenotype, which is related to TENM4 missense mutations, has been identified in humans [107], although contrasting results have been obtained in a study performed in the Canadian population [108]. With regards to TENM4 involvement in chondrocyte muscular differentiation processes, it has been described as a suppressor of chondrogenic differentiation and as a regulator of the molecules involved in chondrogenesis by decreasing Sox6, Sox9, and Runx2 levels and increasing ERK phosphorylation [109]. In the case of satellite cells, also known as muscle stem cells, TENM4 expression has been detected during their quiescent status, but not in proliferating myoblasts. This suggests that TENM4 is down-regulated following the activation and proliferation of satellite cells, probably in response to NOTCH signaling [110], and that TENM4 has a pivotal role in suppressing myogenic differentiation [111]. Overall, these data suggest that TENM4 is involved in cell differentiation. However, since its role is contradictory in tissues of different origins, we can only speculate as to its tissue-specific implications in cell differentiation processes.

TENM4 has also been identified as part of the downstream signaling of the CAAT enhancer binding protein homologous protein (CHOP) [6], a protein that is highly expressed under cellular stress conditions, such as the presence of unfolded proteins in the endoplasmic reticulum. Indeed, TENM4 is expressed upon stress-induced CHOP activation, and then contributes to the activation of the mechanisms that facilitate cellular adaptation to stress [6,112,113].

The importance of TENM4 in neuronal development and cellular differentiation is supported by the evidence of associations between its deregulation and several mental disorders. Indeed, genome-wide association studies have identified TENM4′s involvement in bipolar disorder susceptibly [114], and its role as a risk gene for schizophrenia [115]. Recently, a missense mutation of the gene has been found to co-segregate with schizophrenia, suggesting that TENM4 has a potential role in this pathology [116]. A TENM4 risk variant has also been associated with mood disorders [117], and with the early onset of bipolar disorders [118].

### TENM4 and Cancer

**Figure 5:**
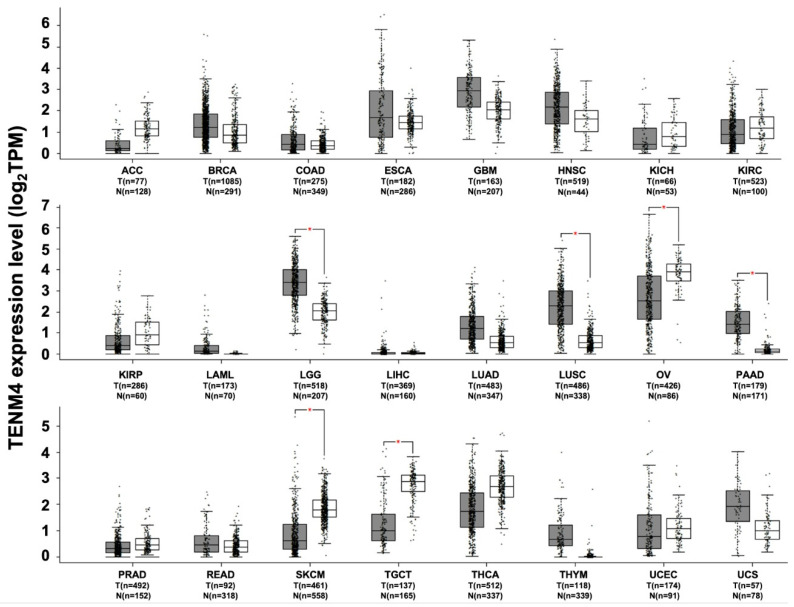
TENM4 expression in different datasets of tumors and normal samples. TENM4 expression level, shown as log2 of transcript per million mapped reads value, was analyzed from RNA sequencing data of tumors and normal samples from the TCGA and the GTEx projects, using a web standard processing pipeline [43]. Tumor (T) and normal samples (N) are represented in grey and white box plots, respectively, each dot representing a sample (*n* = number of samples within the dataset). Statistical differential analysis was performed using the GEPIA default recommended parameters, one-way ANOVA, |Log2FC| ≥1 and *p* ≤ 0.01. Datasets description: ACC; Adrenocortical carcinoma. BRCA; Breast invasive carcinoma. COAD; Colon adenocarcinoma. ESCA; Esophageal carcinoma. GBM; Glioblastoma multiforme. HNSC; Head and neck squamous cell carcinoma. KICH; Kidney chromophobe. KIRC; Kidney renal clear cell carcinoma. KIRP; Kidney renal papillary cell carcinoma. LAML; Acute myeloid leukemia. LGG; Brain lower grade glioma. LIHC; Liver hepatocellular carcinoma. LUAD; Lung adenocarcinoma. LUSC; Lung squamous cell carcinoma. OV; Ovarian serous cystadenocarcinoma. PAAD; Pancreatic adenocarcinoma. PRAD; Prostate adenocarcinoma. READ; Rectum adenocarcinoma. SKCM; Skin cutaneous melanoma. TGCT; Testicular germ cell tumors. THCA; Thyroid carcinoma. THYM; Thymoma. UCEC; Uterine corpus endometrial carcinoma. UCS; Uterine carcinosarcoma.

TENM4 expression has been found to be deregulated in several tumor types. However, few data on the role of TENM4 in carcinogenesis are available. The in silico analysis of RNA sequencing data demonstrates the presence of a significant increase in TENM4 mRNA levels, compared to normal tissues, in brain lower grade glioma, lung adenocarcinoma and pancreatic adenocarcinoma (Figure 5).

A gene expression profile of colon adenocarcinoma has demonstrated the overexpression of TENM4 during cancer progression in a mouse model that mimics human colorectal cancer development [119]. Breast cancer is another tumor type in which TENM4 may have a contribution. Indeed, TENM4 mRNA and protein expression has been found in several human breast cancer cell lines and in breast tumor samples [73]. Moreover, a potential role for TENM4 in breast cancer stem cell self-renewal has been suggested by the recent identification of TENM4 up-regulation in human TNBC stem cell-enriched mammary tumorspheres, compared to parental ones [120,121]. Interestingly, in the breast cancer cell line MDA-MB-175, TENM4 is involved in a translocation that generates the TENM4–neureguilin-1 fusion gene, resulting in ϒ-heregulin fusion protein production [122]. The expression of the fusion protein is controlled by the TENM4 promoter, and it has been shown to induce breast cancer cell proliferation [123] through the constitutive activation of the ErbB3–ErbB2 complex via ϒ-heregulin-dependent autocrine stimulation [123,124]. Indeed, a significant reduction in tumor cell proliferation was observed with the inhibition of ErbB3–ErbB2 complex formation by monoclonal antibodies that targeted ErbB2 [124]. It is worth noting that the ϒ-heregulin fusion protein is not present in normal breast tissues, which indicates that this isoform may be involved in the transformation of normal mammary tissue to breast cancer [124,125]. Nevertheless, the role of ϒ-heregulin in breast cancer proliferation needs to be investigated further, since it has not been found to be expressed in other breast cancer cell lines. The possible contribution of TENM4 to cancer progression is also suggested by the correlation between TENM4 up-regulation and patients’ worst prognosis in endometrial, liver, renal and stomach cancers [35].

A role for TENM4 has also been proposed in the case of ovarian cancer. Interestingly, in this case, significantly lower levels of TENM4 transcripts have been found in the biopsies of undifferentiated mucinous serous ovarian cancer compared to the more differentiated ones. Since cancer cell dedifferentiation is considered a hallmark of aggressiveness, these data suggest that there may be a link between TENM4 expression and patient prognosis. Indeed, a correlation between TENM4 down-regulation and shorter mean survival time was found in patients with serous carcinomas and malignant ovary tumors. Interestingly, TENM4 down-regulation via specific siRNA significantly decreases the sensitivity of ovarian cancer cells to cisplatin [73], suggesting that TENM4 may contribute to ovarian cancer drug resistance. Significantly lower amounts of TENM4 mRNA transcripts, compared to normal tissues, can also be observed in ovarian serous cystadenocarcinoma, in skin cutaneous melanoma and in testicular germ cell tumors (Figure 5). Moreover, TENM4 gene disruption due to HBV genome integration has been frequently observed in hepatocellular carcinomas.

In support of TENM4’s possible contribution to carcinogenesis, several chromosomal rearrangements have been identified in different solid tumors, such as neuroblastoma [126], chronic lymphocytic leukemia [127], small cell lung carcinoma [50], esophageal carcinoma [50], and breast adenocarcinoma [35].

Although contradictory, taken together, these data highlight the involvement of TENM4 in tumor development and progression, and suggest its possible use as a suitable prognostic and predictive biomarker. Indeed, TENM4 peptides have been identified in a proteomic study using human urine [128], and TENM4 was detected as one of the most abundant proteins in the secretome [129] and in the exosomes [130] derived from a neuroblastoma cell line. Although the data on the correlation between TENM4 expression in tumors and its release are still lacking, the possibility of using TENM4 as a disease biomarker, which can be assayed in liquid biopsies obtained from cancer patients, is an interesting field for further investigation.

## 7. Conclusions

Beside the well-documented involvement of teneurins as morphogens and determinants of neural connectivity during embryonic development, accumulating evidence points to their involvement also in carcinogenesis, either as oncogenes or as oncosuppressors, depending on the tumor histotype. Current findings suggest that teneurins deregulation is associated with cancer cells proliferation, migration and invasion. Recently, a possible contribution of teneurins in cancer cell self-renewal [57,59,120,121] and in drug resistance [73] has also been proposed. However, in vivo studies exploiting overexpressing and KO cancer cells are scarce, thus limiting the current knowledge on the precise role of each teneurin in tumorigenesis, and the molecular mechanisms through which each teneurin paralogue contributes to carcinogenesis. Indeed, the high level of complexity of teneurins proteins, and the existence of multiple transcript splice variants leading to possible different tissue-specific regulation mechanisms, make the study of their role in carcinogenesis challenging.

To date, an oncogenic role has been proposed for TENM1 in papillary thyroid carcinoma, prolactin pituitary tumor and glioblastoma. By contrast, a tumor suppressor role has been suggested for TENM2 in ovarian, prostate, cervical cancers and stomach tumors. However, it has to be noted that an oncogenic role for TENM2 cannot be excluded, since in TNBC, a significant correlation between high TENM2 expression and reduced patient metastatic-free survival time has been demonstrated. This dual oncosuppressive and oncogenic role can also be proposed for TENM3. Indeed, it can function as an oncosuppressor in neuroblastoma and as an oncogene in prostate and colorectal cancers, and in glioblastoma. Even if very few data are available for TENM4 role in cancer, decreased expression was found in serous ovarian tumors, whereas TENM4 overexpression has been revealed in colorectal cancer and TNBC, further corroborating the tumor-specific role of teneurins. 

To the best of our knowledge, the molecular mechanisms through which teneurins exert contradictory functions in different tumor histotypes have not been elucidated yet. In particular, no reports are available on how tenurins interact in a given type of cancer, and on the possibility that teneurins’ homo- and/or hetero-dimerization, instead of deregulated expression, could be involved in cancer progression. Moreover, considering that different alternative splice variants can interact with different ligands leading to diverse cellular functions, it can be speculated that these mechanisms could also affect tumorigenesis.

The data mining of publicly available data sets derived from large cohorts of oncologic patients provides interesting evidence about the presence of somatic mutations and chromosomal alterations (e.g., translocations, copy number variations, chromothripsis, and viral genome integration) leading to both teneurins’ inactivation or overexpression in human tumors [35]. Moreover, teneurins’ up-regulation as well as down-regulation has been correlated with tumor progression, metastasization, and patients’ survival [35], suggesting a possible role of teneurins as interesting prognostic biomarkers. The discovery of TENM4 in the human urine [128], in the secretome [129] and in the exosomes [130] of neuroblastoma cell lines might point to a potential role for teneurins as soluble blood and urine tumor biomarkers for non-invasive disease detection and management, at least for the teneurins-overexpressing tumors.

Finally, teneurins could represent a promising targets for cancer therapy, including immunotherapy, in cancer patients that show teneurin overexpression. Clearly, further and more extensive research is needed to explore the feasibility, safety and efficacy of using teneurins as therapeutic targets. Indeed, the expression pattern of teneurins in healthy tissues, and in particular in the nervous system, should be carefully considered when designing an appropriate targeted therapy against teneurins. Most of the information gained so far on the biological role of teneurins regards developmental processes; therefore, the targeting of teneurins in adult tissues could have no dramatic effects in the physiology of fully developed organs and tissues.

## Data Availability

Not Applicable.
